# Reemergence of Epidemic *Vibrio cholerae* O139, Bangladesh

**DOI:** 10.3201/eid0909.020443

**Published:** 2003-09

**Authors:** Shah M. Faruque, Nityananda Chowdhury, M. Kamruzzaman, Q. Shafi Ahmad, A.S.G. Faruque, M. Abdus Salam, T. Ramamurthy, G. Balakrish Nair, Andrej Weintraub, David A. Sack

**Affiliations:** * International Centre for Diarrhoeal Disease Research, Dhaka, Bangladesh; † National Institute of Cholera and Enteric Diseases, Beliaghata, Calcutta, India; ‡ Karolinska Instute, Huddinge, Sweden

**Keywords:** Cholera epidemic, *Vibrio cholerae* O139, CTX phage, drug resistance, Bangladesh

## Abstract

During March and April 2002, a resurgence of *Vibrio cholerae* O139 occurred in Dhaka and adjoining areas of Bangladesh with an estimated 30,000 cases of cholera. Patients infected with O139 strains were much older than those infected with O1 strains (p<0.001). The reemerged O139 strains belong to a single ribotype corresponding to one of two ribotypes that caused the initial O139 outbreak in 1993. Unlike the strains of 1993, the recent strains are susceptible to trimethoprim, sulphamethoxazole, and streptomycin but resistant to nalidixic acid. The new O139 strains carry a copy of the Calcutta type CTX^Calc^ prophage in addition to the CTX^ET^ prophage carried by the previous strains. Thus, the O139 strains continue to evolve, and the adult population continues to be more susceptible to O139 cholera, which suggests a lack of adequate immunity against this serogroup. These findings emphasize the need for continuous monitoring of the new epidemic strains.

*Vibrio cholerae* O139 Bengal first emerged during 1992 and 1993 and caused large epidemics of cholera in Bangladesh, India, and neighboring countries (*1*–*3*). This new strain initially displaced the existing *V. cholerae* O1 strains. During 1994 to the middle of 1995, in most northern and central areas of Bangladesh, the O139 vibrios were replaced by a new clone of *V. cholerae* O1 of the El Tor biotype, whereas in the southern coastal regions the O139 vibrios continued to exist (*4*–*6*). During late 1995 and 1996, cases of cholera attributable to both *V. cholerae* O1 and O139 were again detected in various regions of Bangladesh. Since 1996, cholera in Bangladesh has been caused mostly by *V. cholerae* O1 of the El Tor biotype; only a few cases have been attributable to O139 serogroup strains. The epidemiology of cholera in Bangladesh changed again recently, and a large outbreak of cholera caused predominantly by *V. cholerae* O139 occurred in the capital city Dhaka and adjoining areas.

From early March to the end of April 2002, approximately 2,350 cholera patients associated with *V. cholerae* O139 were admitted to the Dhaka Hospital of the International Centre for Diarrhoeal Disease Research, Bangladesh (ICDDR,B). A preliminary estimate showed that >30,000 cases of cholera occurred in Dhaka and the adjoining areas during this outbreak (A.S.G. Faruque, unpub. data). Since the initial emergence of *V. cholerae* O139 in 1992, we have monitored cholera outbreaks caused by this serogroup in Bangladesh and neighboring regions and have conducted several studies to characterize O139 strains. These studies indicate that strains of the O139 serogroup are undergoing rapid genetic changes, resulting in the origination of new clones; at least seven different ribotypes of O139 vibrios have been documented (*6*–*8*). Furthermore, O139 vibrios may have originated from more than one progenitor strain (*8*). The transient disappearance and reemergence of *V. cholerae* O139 in Bangladesh have raised questions regarding the origin of the reemerged O139 vibrios. In this study, we examined the current epidemiology of cholera in Bangladesh and analyzed *V. cholerae* O139 isolated from the recent outbreak to investigate the origin of the recent epidemic strains as well as to characterize possible genetic changes in O139 vibrios that might have contributed to the recent resurgence of *V. cholerae* O139.

## Materials and Methods

### Clinical Surveillance

ICDDR,B maintains a 2% surveillance system at its Dhaka Hospital, in which data from every 50th patient treated at the hospital is collected; these data include clinical information and biologic specimens. We used these data to extrapolate the overall numbers of patients with cholera; specimens from these patients were used in the bacteriologic studies described.

### *V. cholerae* Strains

A total of 63 *V. cholerae* O139 isolates obtained from the recent cholera epidemic were analyzed. Seven strains of O139 vibrios isolated in India between 1992 and 1996, 17 strains of *V. cholerae* O139 isolated in Bangladesh between 1993 and 1997, and 2 strains isolated in Thailand in 1998 were also included in the study for comparison with the recent epidemic strains. Strains of the recent epidemic were isolated from stools of cholera patients who attended the treatment center of ICDDR,B located in Dhaka during March and April 2002. Stool samples were processed in the laboratory within 2 h of collection for the isolation of *V. cholerae*. Stools were initially streaked on thiosulphate-citrate-bile-sucrose (Becton, Dickinson and Co., Sparks, MD) agar plates for selection and presumptive identification of *V. cholerae*. All strains were subsequently examined by biochemical and serologic tests using standard methods (*9*). Strains were stored in sealed deep nutrient agar at room temperature until used for this study. Details of the strains are shown in Table.

### Polymerase Chain Reaction (PCR) Assays

Presence of *tcpA* genes specific for the classical and El Tor biotypes was determined by using a multiplex PCR assay, as described previously (*10*). PCR assays for the *tcpI* and *acfB* genes have been described previously (*6*). Presence of classical, El Tor, and Calcutta type *rstR* genes of CTX phage were also determined with PCR by using specific primers derived from the published sequence of the respective genes. Three different forward primers for rstR^class^, rstR^ET^, and rstR^Calc^ with sequences 5′-CTTCTCATCAGCAAAGCCTCCATC, 5′-GCACCATGATTTAAGATGCTC, and 5′-CTGTAAATCTCTTCAATCCTAGG, respectively, were used with a common reverse primer (5′-TCGAGTTGTAATTCATCAAGAGTG) to amplify the respective rstR genes. Presence of the *rstC* gene was also determined by a PCR assay described previously (*11*). All primers were synthesized commercially by Oswel DNA Service (University of Edinburgh, Edinburgh, UK). The expected sizes of the amplicons were ascertained by electrophoresis in agarose gels, and the identity of each PCR product was further verified by Southern blot hybridization.

### Probes and Hybridization

The gene probes used in this study included a 0.5 kb *Eco*RI fragment of pCVD27 (*12*) containing part of the *ctxA* gene and a 2.1 kb *Sph*I-*Xba*I fragment of pCTX-Km containing the entire *zot* and *ace* genes and part of *orfU* (*13*). The *toxR* gene probe was a 2.4-kb *Bam*HI fragment of pVM7 (*14*). The *rstR*^ET^ probe was a *Sac*I-*Xba*I fragment of pHK1 (*15*). The rRNA gene probe was a 7.5-kb *Bam*HI fragment of the *Escherichia coli* rRNA clone pKK3535 described previously (*16*). The O139-specific DNA probe 2R3 was a 1.3-kb *Eco*RI fragment of pCRII-A3 (*17*,*18*), and the SXT probe was a *Not*I fragment of pSXT1 (*19*). PCR-generated amplicons of the *rstR* genes of classical, El Tor, or Calcutta type CTX prophage were also used as probes whenever appropriate.

For preparation of Southern blots, total cellular DNA was isolated from overnight cultures as described previously (*20*). Five-microgram aliquots of the DNA were digested with appropriate restriction enzymes (Bethesda Research Laboratories, Gaithersburg, MD), electrophoresed in 0.8% agarose gels, blotted onto nylon membranes (Hybond, Amersham Biosciences, Uppsala, Sweden), and processed by using standard methods (*21*,*22*). The probes were labeled by random priming (*23*) using a DNA labeling kit (Bethesda Research Laboratories) and α-^32^P‑deoxycytidine triphosphate (3,000 Ci/mmol, Amersham Biosciences). Southern blots were hybridized with the labeled probes at 68°C and washed under stringent conditions as described previously (*6*,*8*). Autoradiographs were developed from the hybridized filters with Kodak X‑Omat AR x‑ray film (Eastman Kodak Co., Rochester, NY) at –70°C.

### Antimicrobial Resistance

All *V. cholerae* isolates were tested for resistance to antimicrobial drugs by using the method of Bauer et al. (*24*) with standard antibiotic disks (Oxoid Ltd., Basingstoke, Hampshire, UK) at the following antibiotic concentrations (μg/disc): ampicillin, 10; chloramphenicol, 30; streptomycin, 10; tetracycline, 30; trimethoprim‑sulfamethoxazole, 1.25 and 23.75, respectively; kanamycin, 30; gentamicin, 10; ciprofloxcin 5; norfloxacin 10, and nalidixic acid, 30.

## Results

### Clinical Surveillance

**Figure 1:**
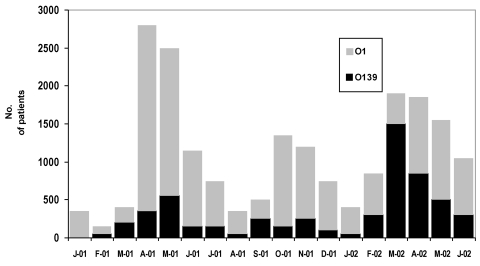
Numbers of diarrhea patients with cholera attributed to *Vibrio cholerae* O1 and O139 from January 2001 to June 2002. Those positive for cholera are extrapolated from a 2% sample of all patients receiving treatment.

We noted a marked increase in cholera cases associated with *V. cholerae* O139 from March to May 2002 (Figure 1). The highest number of cholera patients admitted to the hospital was in March; 69.8% of these cases were attributed to *V. cholerae* O139, compared to 30.2% of cases caused by the El Tor biotype of *V. cholerae* O1. Cholera attributable to *V. cholerae* O139 occurred with similar frequencies in men and women, similar to those infected with O1 strains. From January 2001 to June 2002, a total of 91 (32%) of 282 of case-patients infected with O1 cholera were <5 years of age, but 15 (15%) of 115 of those infected with O139 were <5 years of age (p<0.001). During the same period, 48% of those infected with *V.* *cholerae* O1 were >15 years of age, while 76% of those infected with O139 were >15 years of age (p<0.001).

### Genetic Analysis of *V. cholerae* Strains

**Figure 2:**
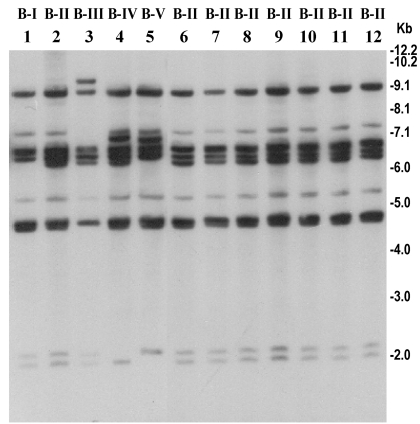
Southern hybridization analysis of rRNA genes in *Vibrio cholerae* O139 strains isolated from the recent epidemic and comparison with representative O139 strains isolated between 1992 and 1998. Genomic DNA was digested with *Bgl*I and probed with a 7.5-kb *Bam*HI fragment of the *Escherichia coli* rRNA clone pKK3535. Lanes 1–6 represent O139 strains isolated from 1992 to 1998; lanes 6–12 represent O139 strains isolated from the recent epidemic in Bangladesh. Designated ribotypes corresponding to each restriction pattern are shown on top of the corresponding lane. Numbers indicating molecular sizes of bands correspond to 1-kb DNA ladder (Bethesda Research Laboratories, Bethesda, MD) used as molecular size markers.

**Figure 3:**
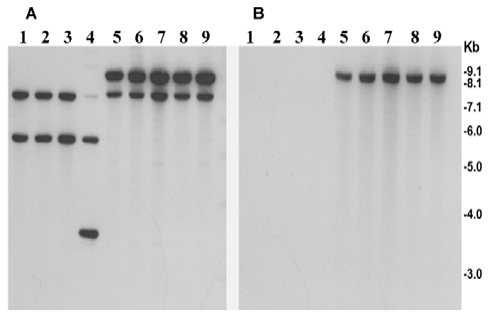
Southern hybridization analysis of *rstR* genes in toxigenic *Vibrio cholerae* O139 strains isolated from the recent epidemic in Bangladesh (lanes 5–9) and in previously isolated O139 strains from 1992 to 1998 (lanes 1–4). Genomic DNA was digested with *Bgl*I and probed with the rstR^ET^ probe (A) and with the rstR^Cal^ probe (B). Numbers indicating molecular sizes of bands correspond to 1-kb DNA ladder (BRL) used as molecular size markers.

The rRNA gene restriction patterns using *Bgl*I consisted of 10 to 14 bands between 11 kb and 1.6 kb in size (Figure 2). The 89 analyzed strains belonged to four different ribotypes (B-I to B-IV). All 63 recently isolated O139 strains produced identical restriction patterns of their rRNA genes and belonged to ribotype B-II. Analysis of the *rstR* gene showed that O139 strains isolated from 1992 to 1998 carried El Tor type CTX^ET^ prophage, whereas the recent epidemic strains carry the Calcutta type CTX^Calc^ prophage in addition to the CTX^ET^ prophage (Figure 3). All strains were positive for *tcpA*, *tcpI*, *acfB*, *toxT*, *ctxA*, *zot*, and *ToxR* genes, as well as for the O139-specific genomic DNA in DNA probe or PCR assays.

### Antibiogram

All strains isolated from the recent epidemic were resistant to nalidixic acid and were susceptible to ampicillin, tetracycline, gentamicin, chloramphenicol, ciprofloxacin, norfloxacin, streptomycin, trimethoprim, and sulfamethoxazole. In these strains, the SXT element, which encodes resistance to streptomycin, sulfamethoxazole, and trimethoprim, carried a deletion of an approximately 3.6-kb region.

## Discussion

Generally, a seasonality exists in the cholera cases seen at the ICDDR,B hospital, with increased numbers expected before and after the rainy season. Thus, the increase in total number of cases seen during March and April was not unusual (Figure 1). However, the increase in patient numbers during these months of 2002 was associated with a marked increase in cases associated with *V. cholerae* O139, and the numbers of cases infected with serogroup O139 outnumbered those with serogroup O1. The ages of patients infected with O139 strains were significantly higher than those infected with O1 strains (p<0.001). Since the onset of O139 cholera in 1992, this organism has tended to infect patients older than those with O1 cholera (*1*). The more advanced age of this group was explained by a lack of immunity to this new serogroup in adults who were likely partially immune to the O1 serogroup. Thus, after nearly 10 years of endemicity in Bangladesh, *V. cholerae* O139 continues to cause more cases of cholera in older adults.

### Ribotype Analysis

The emergence of the O139 serogroup has provided a unique opportunity to witness the epidemiologic changes associated with the displacement of an existing serogroup by a new emerging one and thus provides new insights into the epidemiology of the disease. All 63 recently isolated O139 strains produced the identical restriction pattern of their rRNA genes. This restriction pattern has been previously designated as ribotype pattern B-II (*6*–*8*) and was first detected among epidemic *V. cholerae* O139 strains that emerged in 1992 and 1993. Cholera epidemics during 1992 to 1993 in India and Bangladesh that were associated with the first appearance of *V. cholerae* O139 were caused by strains belonging to two different ribotypes, designated as B-I and B-II. Since then, several new ribotypes of O139 vibrios have been detected which were associated with localized outbreaks during 1995 to 1996 or sporadic cases (*8*). The results suggest that strains of the recent epidemic were clonal and were derived from one of the initial clones of *V. cholerae* O139. We therefore investigated possible genetic changes sustained by this strain during the nearly 9 years since major epidemics were caused by strains of this ribotype.

### Analysis of CTX Prophage

In *V. cholerae*, the genes encoding cholera toxin (*ctxAB*) are part of the CTX prophage (*25*). A typical CTXΦ genome has two regions: core and the RS2. The 4.5-kb core region comprises several open reading frames including *ctxAB*, *zot*, *ace*, *orfU*, and encodes CT as well as the functions that are required for the virion morphogenesis; by contrast, the 2.5-kb RS2 region encodes the regulation, replication, and integration functions of the CTXΦ genome (*26*). Previous studies have described the existence of at least three widely diverse repressor genes (*rstR* genes) carried by different CTX phages (i.e., CTX^ET^Φ, CTX^class^Φ, and CTX^Calc^Φ) (*27*,*28*). This diversity of *rstR* constitutes the molecular basis for heteroimmunity among CTX phages, which are otherwise genetically similar. We examined the CTX prophage in the recent and previously isolated O139 strains with specific probes. Analysis of the *rstR* gene carried by the recent epidemic strains showed that, unlike the O139 strains of 1993, which carried multiple copies of an El Tor type CTX_ET_ prophage, the new O139 strains carry at least one copy of the Calcutta type CTX^Calc^ prophage in addition to the CTX^ET^ prophage. As a result of heteroimmunity, toxigenic classical strains of *V. cholerae* O1 are known to be infected by CTXΦ isolated from El Tor biotype strains; toxigenic El Tor strains are resistant to further infection by the same phage. Similarly, strains carrying an El Tor type CTX prophage can be superinfected by the Calcutta type CTX phage (*29*). Therefore, the new epidemic strains appear to have arisen by acquisition of a Calcutta type CTX phage by strains that originally harbored only El Tor type CTX prophage, since the new strains carry both prophages (Figure 3). What determines the reemergence of particular epidemic strains is not clear, but this study clearly shows changes in the CTX genotype attributed to the acquisition of a new CTX phage by the O139 strains associated with the recent epidemic.

### Antibiogram of Reemergent O139 Strains

**Figure 4:**
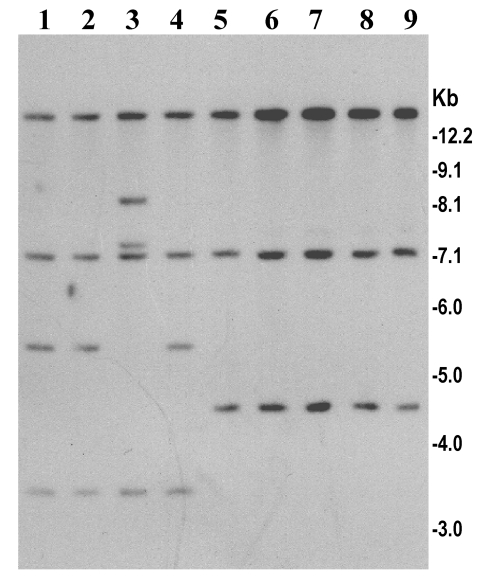
Analysis of SXT element in *V. cholerae* O139 strains isolated isolated from the recent epidemic in Bangladesh (lanes 5–9) and in previously isolated O139 strains between 1992 and 1998 (lanes 1–4). Genomic DNA was digested with *Bgl*I and probed with the SXT gene probe. Lanes 1, 2, and 4 show restriction pattern corresponding to SXT genotype A, lanes 3 shows SXT genotype B, and lanes 5–9 represent SXT genotype C. Numbers indicating molecular sizes of bands correspond to 1-kb DNA ladder (BRL).

*V. cholerae* O139, which emerged during 1992 and 1993, was sensitive to tetracycline and showed a trend of increased resistance to trimethoprim-sulfamethoxazole (SXT) and streptomycin. This resistance was mediated by a ~99-kb self-transmissible transposon-like element (SXT constin) encoding resistance to sulfamethoxazole, trimethoprim, and streptomycin, the resistance genes being clustered together in a 9.4-kb region (*19*). In the present study, all strains isolated from the recent epidemic were found to be susceptible to SXT and streptomycin (Table). To identify the genetic changes associated with the observed SXT sensitivity, we used a cloned SXT gene probe to study restriction fragment length polymorphism in the SXT transposon. Three different *Bgl*I restriction patterns (patterns A–C) of the SXT element were observed among the O139 strains tested (Figure 4). Strains producing pattern A and B were resistant to SXT and streptomycin and included strains isolated between 1992 and 1996, whereas all strains from the recent epidemic produced pattern C and were susceptible to all the three antibiotics. Further analysis of the restriction patterns suggests that the restriction site heterogeneity possibly occurred as a result of a deletion of approximately a 3.6-kb region of the SXT element in strains that were sensitive to SXT and streptomycin. The deletion in the SXT element associated with sensitivity to SXT and streptomycin was first detected in strains of ribotype B-III isolated from an outbreak in Bangladesh in 1997 (*6*). In keeping with the observation in Bangladesh, comparison of the antibiotic resistance patterns between the O139 strains isolated during 1992 and 1993 and those isolated in 1996 and 1997 in India also showed that the later strains were susceptible to SXT, unlike the O139 strains from 1992 and 1993 (*30*). However, in contrast to the previously isolated O139 strains, all O139 strains isolated from the recent epidemic were resistant to nalidixic acid.

**Table:** Comparative analysis of 63 *Vibrio cholerae* O139 strains isolated from recent epidemic in Bangladesh versus O139 strains isolated between 1993 and 1998 in different countries

Y of isolation	Country	No. of isolates	Ribotype^a^	Presence of genes^b^				STX genotype^a^	Antibiogram^c^
				*rstR^ET^*	*rstR^Clas^*	*rstR^Cal^*	*rstC*		
1993	Bangladesh	5	B-I	+	-	-	+	A	S^R^, SXT^R^
1993	Bangladesh	1	B-II	+	-	-	+	B	S^R^, SXT^R^
1993–1995	Bangladesh	5	B-II	+	-	-	+	A	S^R^, SXT^R^
1997	Bangladesh	3	B-II	+	-	-	+	C	Susceptible^b^
1997	Bangladesh	3	B-III	+	-	-	+	C	Susceptible^b^
1992	India	3	B-I	+	-	-	+	A	S^R^, SXT^R^
1993	India	1	B-V	+	-	-	+	A	A^R^, S^R^, SXT^R^
1994	India	1	B-IV	+	-	-	+	A	S^R^, SXT^R^
1996	India	2	B-II	+	-	-	+	A	A^R^, Fz^R^, S^R^, SXT^R^
1998	Thailand	2	B-I	+	-	-	+	A	S^R^, SXT^R^
2002	Bangladesh	63	B-II	+	-	+	+	C	Nal^R^

^a^ Ribotypes and SXT genotypes are based on *Bgl*I restriction patterns of the respective genes and their flanking chromosomal sequence. ^b^All strains were positive for *tcpA*, *tcpI*, *acfB*, *toxT*, *ctxA*, *zot*, and *ToxR* genes as well as for the O139-specific genomic DNA in DNA probe or polymerase chain reaction assays. ^c^All strains were susceptible to tetracycline, ampicillin, chloramphenicol, gentamicin, ciprofloxacin, norfloxacin, nalidixic acid, streptomycin, trimethoprim, and sulfamethoxazole.

### Epidemiologic Importance of Genetic Changes in *V. cholerae* O139

Several previous studies have shown that the O139 serogroup of *V. cholerae* has been undergoing rapid genetic changes (*6*–*8*) since its first emergence. We speculate that the observed changes may have provided increased fitness to strains of this serogroup in some unexplained way to survive in competition with the existing seventh pandemic strain of *V. cholerae* O1 and establish itself as the etiologic agent of a possible eighth pandemic. The transient disappearance of the O139 serogroup in Bangladesh and repeated reemergence associated with somewhat altered genetic or phenotypic properties seem to support this speculation. Our study demonstrated the reemergence of *V. cholerae* O139 strains belonging to a previously described ribotype which has sustained at least three major genetic and phenotypic changes. These changes include the acquisition of a new CTX prophage, deletion in the SXT element associated with reversion of drug resistance phenotype against SXT and streptomycin, and development of nalidixic acid resistance.

The recent epidemic strains were otherwise similar to previously described O139 strains, including possession of the TCP pathogenicity island, as evidenced by the presence of *tcpA*, *tcpI*, and *acfB* genes; the virulence regulatory genes, *toxT* and *toxR*; and the O139–serotype-specific DNA. The role of environmental and host factors that contribute to the emergence of new strains associated with epidemic outbreaks is not clearly known. In the present study, all strains isolated from the recent cholera outbreak belonged to the same ribotype and were genetically and phenotypically identical, suggesting that the recent outbreak in Bangladesh probably started from a point source and may have coincided with the acquisition of one or more critical new properties by a previously existing *V. cholerae* O139 strain. Clearly these properties included the acquisition of the Calcutta Type CTX prophage. Previous studies showed that O139 strains prevailing in Calcutta during 1996 carried this prophage (*29*,*31*,*32*), which might have contributed to the dissimilar incidence of O139 cholera in Calcutta and Dhaka during that period (*33*). How the initial enrichment of *V. cholerae* occurred before the initiation of an epidemic is not clear. We speculate that a critical factor for the recent reemergence of O139 vibrios might have been the development of nalidixc acid resistance. Identifying the first index case of the present cholera epidemic is not possible. A spontaneous nalidixic acid–resistant *V. cholerae* O139 strain may have been enriched in a patient undergoing nalidixc acid therapy, leading to the eventual spread of the organism. This is certainly possible in view of the widespread use of nalidixic acid in Bangladesh as a drug to treat other gastroenteritis, including shigellosis. The emergence of *V. cholerae* O139 has received global attention not only as the first non–O1 *V. cholerae* capable of causing epidemic outbreaks but also because of the rapid genetic re-assortment undergone by strains of this new serogroup. Our study shows yet another set of genetic and phenotypic changes in O139 vibrios and their association with an epidemic of cholera in Bangladesh. These results emphasize the need for continuing molecular epidemiologic surveillance of *V. cholerae* in Bangladesh and adjoining areas.

## References

[1] Cholera Working Group, International Centre of Diarrhoeal Disease Research, Bangladesh. Large epidemic of cholera like disease in Bangladesh caused by *Vibrio cholerae* O139 synonym Bengal. Lancet. 1993;342:387–90. 10.1016/0140-6736(93)92811-7 8101899

[2] Ramamurthy T, Garg S, Sharma R, Bhattacharya SK, Nair GB, Shimada T, Emergence of a novel strain of *Vibrio cholerae* with epidemic potential in Southern and Eastern India. Lancet. 1993;341:703–4. 10.1016/0140-6736(93)90480-5 8095620

[3] Faruque SM, Albert MJ, Mekalanos JJ. Epidemiology, genetics and ecology of toxigenic *Vibrio cholerae.* Microbiol Mol Biol Rev. 1998;62:1301–14. 9841673 10.1128/mmbr.62.4.1301-1314.1998 PMC98947

[4] Faruque ASG, Fuchs GJ, Albert MJ. Changing epidemiology of cholera due to *Vibrio cholerae* O1 and O139 Bengal in Dhaka, Bangladesh. Epidemiol Infect. 1996;116:275–8. 10.1017/S0950268800052572 8666070 PMC2271418

[5] Faruque SM, Ahmed KM, Alim ARMA, Qadri F, Siddique AK, Albert MJ. Emergence of a new clone of toxigenic *Vibrio cholerae* biotype El Tor displacing *V. cholerae* O139 Bengal in Bangladesh. J Clin Microbiol. 1997;35:624–30. 9041401 10.1128/jcm.35.3.624-630.1997 PMC229639

[6] Faruque SM, Siddique AK, Saha MN, Asadulghani, Rahman MM, Zaman K, et al. Molecular characterization of a new ribotype of *Vibrio cholerae* O139 Bengal associated with an outbreak of cholera in Bangladesh. J Clin Microbiol. 1999;37:1313–8. 10203477 10.1128/jcm.37.5.1313-1318.1999 PMC84761

[7] Faruque SM, Saha MN, Asadulghani, Bag PK, Bhadra PK, Bhattacharya SK,et al. Genomic diversity among *Vibrio cholerae* O139 strains isolated in Bangladesh and India between 1992 and 1998. FEMS Microbiol Lett. 2000;184:279–84. 10.1111/j.1574-6968.2000.tb09027.x 10713434

[8] Faruque SM, Saha MN, Asadulghani, Sack DA, Sack RB, Takeda Y, et al. The O139 serogroup of *Vibrio cholerae* comprises diverse clones of epidemic and nonepidemic strains derived from multiple *V. cholerae* O1 and non-O1 progenitors. J Infect Dis. 2000;182:1161–8. 10.1086/315807 10979913

[9] World Health Organization. World Health Organization guidelines for the laboratory diagnosis of cholera. Geneva: The Organization; 1974.

[10] Keasler SP, Hall RH. Detection and biotyping *Vibrio cholerae* O1 with multiplex polymerase chain reaction. Lancet. 1993;341:1661. 10.1016/0140-6736(93)90792-F 8100020

[11] Faruque SM, Asadulghani, Kamruzzaman M, Nandi RK, Ghosh AN, Nair GB, et al. RS1 element of *Vibrio cholerae* can propagate horizontally as a filamentous phage exploiting the morphogenesis genes of CTXΦ. Infect Immun. 2002;70:163–70. 10.1128/IAI.70.1.163-170.2002 11748178 PMC127613

[12] Kaper JB, Morris JG Jr, Nishibuchi M. DNA probes for pathogenic *Vibrio* species. In: Tenover FC, editor. DNA probes for infectious disease. Boca Raton (FL): CRC Press, Inc.; 1988. p. 65–77.

[13] Faruque SM, Asadulghani, Saha MN, Alim ARMA, Albert MJ, Islam KMN, et al. Analysis of clinical and environmental strains of nontoxigenic *Vibrio cholerae* for susceptibility to CTXΦ: molecular basis for the origination of new strains with epidemic potential. Infect Immun. 1998;66:5819–25. 9826360 10.1128/iai.66.12.5819-5825.1998 PMC108736

[14] Miller VL, Mekalanos JJ. Synthesis of cholera toxin is positively regulated at the transcriptional level by *toxR.* Proc Natl Acad Sci U S A. 1984;81:3471–5. 10.1073/pnas.81.11.3471 6374658 PMC345530

[15] Kimsey HH, Waldor MK. CTXΦ immunity: application in the development of cholera vaccines. Proc Natl Acad Sci U S A. 1998;95:7035–9. 10.1073/pnas.95.12.7035 9618534 PMC22729

[16] Brosius J, Ullrich A, Raker MA, Gray A, Dull TJ, Gutell RR, Construction and fine mapping of recombinant plasmids containing the rrnB ribosomal RNA operon of *E. coli.* Plasmid. 1981;6:112–8. 10.1016/0147-619X(81)90058-5 7025054

[17] Nair GB, Bag PK, Shimada T, Ramamurthy T, Takeda T, Yamamoto S, Evaluation of DNA probes for specific detection of *Vibrio cholerae* O139 Bengal. J Clin Microbiol. 1995;33:2186–7. 7559975 10.1128/jcm.33.8.2186-2187.1995 PMC228362

[18] Waldor MK, Mekalanos JJ. *Vibrio cholerae* O139 specific gene sequence. Lancet. 1994;343:1366. 10.1016/S0140-6736(94)92504-6 7910357

[19] Hochhut B, Lotfi Y, Mazel D, Faruque SM, Woodgate R, Waldor MK. Molecular analysis of antibiotic resistance gene clusters in *Vibrio cholerae* O139 and O1 SXT constins. Antimicrob Agents Chemother. 2002;45:2991–3000. 10.1128/AAC.45.11.2991-3000.2001 11600347 PMC90773

[20] Stull TL. LiPuma JJ, Edlind TD. A broad spectrum probe for molecular epidemiology of bacteria: ribosomal RNA. J Infect Dis. 1988;157:280–6. 2447202 10.1093/infdis/157.2.280

[21] Maniatis T, Fritsch EF, Sambrook J. Molecular cloning: a laboratory manual. Cold Spring Harbor (NY): Cold Spring Harbor Laboratory; 1982.

[22] Southern EM. Detection of specific sequences among DNA fragments separated by gel electrophoresis. J Mol Biol. 1975;98:503–17. 10.1016/S0022-2836(75)80083-0 1195397

[23] Feinberg A, Volgelstein B. A technique for radio labelling DNA restriction endonuclease fragments to high specific activity. Anal Biochem. 1984;137:266–7. 10.1016/0003-2697(84)90381-6 6329026

[24] Bauer AW, Kirby WMM, Sherris JC, Turk M. Antibiotic susceptibility by a standardized single disk method. Am J Clin Pathol. 1966;45:493–6. 5325707

[25] Waldor MK, Mekalanos JJ. Lysogenic conversion by a filamentous phage encoding cholera toxin. Science. 1996;272:1910–4. 10.1126/science.272.5270.1910 8658163

[26] Waldor MK, Rubin EJ, Gregory DN, Kimsey HH, Makalanos JJ. Regulation, replication and integration functions of the *Vibrio cholerae* CTXΦ are encoded by region RS2. Mol Microbiol. 1997;24:917–26. 10.1046/j.1365-2958.1997.3911758.x 9220000

[27] Boyd EF, Heilpern AJ, Waldor MK. Molecular analysis of a putative CTXΦ precursor and evidence for independent acquisition of distinct CTXΦs by toxigenic *Vibrio cholerae.* J Bacteriol. 2000;182:5530–8. 10.1128/JB.182.19.5530-5538.2000 10986258 PMC110998

[28] Davis BM, Moyer KE, Boyd EF, Waldor MK. CTX prophages in classical biotype of *Vibrio cholerae*: functional phage genes but dysfunctional phage genomes. J Bacteriol. 2000;182:6992–8. 10.1128/JB.182.24.6992-6998.2000 11092860 PMC94825

[29] Davis BM, Kimsey HH, Chang W, Waldor MK. The *Vibrio cholerae* O139 Calcutta bacteriophage CTXΦ is infectious and encodes a novel repressor. J Bacteriol. 1999;181:6779–87. 10542181 10.1128/jb.181.21.6779-6787.1999 PMC94144

[30] Mitra R, Basu A, Dutta D, Nair GB, Takeda Y. Resurgence of *Vibrio cholerae* O139 Bengal with altered antibiogram in Calcutta, India. Lancet. 1996;348:1181. 10.1016/S0140-6736(05)65326-3 8888210

[31] Sharma C, Maiti S, Mukhopadhyay AK, Basu A, Nair GB, Mukhopadhyaya R, Unique organization of the CTX genetic element in *Vibrio cholerae* O139 strains which reemerged in Calcutta, India, in September, 1996. J Clin Microbiol. 1997;35:3348–50. 9399556 10.1128/jcm.35.12.3348-3350.1997 PMC230184

[32] Kimsey HH, Nair GB, Ghosh A, Waldor MK. Diverse CTXΦ and evolution of new pathogenic *Vibrio cholerae.* Lancet. 1998;352:457–8. 10.1016/S0140-6736(05)79193-5 9708764

[33] Basu A, Mukhopadhyay AK, Sharma C, Jyot J, Gupta N, Ghosh A, Heterogeneity in the organization of the CTX genetic element in strains of *Vibrio cholerae* O139 Bengal isolated from Calcutta, India and Dhaka, Bangladesh and its possible link to the dissimilar incidence of O139 cholera in the two locales. Microb Pathog. 1998;24:175–83. 10.1006/mpat.1997.0186 9514639

